# A Review of Selected Genes with Known Effects on Performance and Health of Cattle

**DOI:** 10.3389/fvets.2016.00113

**Published:** 2016-12-15

**Authors:** Eduardo Casas, Marcus E. Kehrli

**Affiliations:** ^1^National Animal Disease Center, USDA, ARS, Ames, IA, USA

**Keywords:** genes, cattle, genetic conditions, beef, dairy, genomics

## Abstract

There are genetic conditions that influence production in dairy and beef cattle. The objective of this review was to describe relevant genetic conditions that have been associated with productivity and health in cattle. Genes or genomic regions that have been identified as a candidate for the condition will be included, and the genetic basis of the condition will be defined. Genes and genetic conditions included in this review are bovine leukocyte adhesion deficiency, deficiency of the uridine monophosphate synthase, bovine chronic interstitial nephritis, horn development, myostatin, complex vertebral malformation, leptin, osteopetrosis, apoptosis peptide activating factor 1, chondrodysplastic dwarfism, caseins, calpastatin, umbilical hernia, lactoglobulin, citrullinemia, cholesterol deficiency, prions, thyroglobulin, diacylglycerol acyltransferase, syndactyly, maple syrup urine disease, slick hair, Factor XI deficiency, and μ-Calpain. This review is not meant to be comprehensive, and relevant information is provided to ascertain genetic markers associated with the conditions.

## Introduction

Some traits or genetic conditions are controlled by a single gene (monogenic or qualitative traits), while others are controlled by many genes (polygenic or quantitative traits). Eighty-seven percent of qualitative traits in cattle are recessively inherited (1). It is not surprising that genetic conditions are breed specific, given that cattle breeds were developed in relative genetic isolation and independently of each other (1). Until the advent of modern molecular biology methods, the technology was unavailable to identify genes associated with quantitative traits and the variants within the gene that produce differences in productivity or its expression.

Genome-wide association studies (GWASs) are possible due to the availability of technology that allows high-throughput genotyping of single-nucleotide polymorphisms (SNPs). These SNPs are variants, or alleles, in the DNA sequence that may be associated with the expression of a trait or characteristic in cattle. The technology allows deciphering the genetics behind the expression of economically important traits. Genomic regions associated with productive traits have been identified in dairy (2–4) and beef cattle (5–8). It has also been possible to identify genomic regions associated with infectious and genetic diseases that affect performance in cattle (9, 10).

High-throughput sequencing offers the opportunity to identify causative genetic variants, which was previously unavailable (11). Accessibility of this technology has allowed the identification of unknown variants that could possibly be responsible for the conditions. This is particularly important when a condition has been reported in several breeds, and the putative causative mutation has been identified in one breed but not in another. It could be possible that additional variants in the same gene have similar effect but are unidentified. Such is the case of double muscling in cattle (12, 13). This technology has been successfully used to identify genetic variants that cause differences in productivity of milk (14, 15), muscling (15, 16), and fertility (17). Combining GWAS with high-throughput sequencing procedures will enable the identification of unknown causative genetic variants that could improve productivity of cattle.

Several genes have been identified as having an association with productivity and health-related traits in cattle. This review is not meant to be a comprehensive list of genes, given that science continually discovers the association of genes with economically important traits. This review is meant to provide information on relevant conditions that could potentially impact the productivity and health of the cattle industry, and not to detail modifications in the genome that results in each condition. This is because several conditions are produced by different variants in the DNA sequence, as it is the case for double muscling (*MSTN*) in beef cattle where different SNP and insertion/deletions are responsible for the condition (18, 19). For simplicity, this review was organized by bovine chromosomes, rather than by the influence of the gene in productivity or health of cattle. Relevant conditions will be discussed. Table 1 summarizes the genetic conditions discussed in this review.

**Table 1:** **Selected genes with known effects on performance and health of cattle**.

Condition	Chromosome	Locus	Gene
Bovine leukocyte adhesion deficiency	BTA1	BLAD	*CD18*
Deficiency of the uridine monophosphate synthase	BTA1	DUMPS	*UMPS*
Bovine chronic interstitial nephritis	BTA1	CINF	*PCLN1/CL16*
Horn development	BTA1	POLL	–
Myostatin	BTA2	mh	*MSTN*
Complex vertebral malformation	BTA3	CVM	*SLC35A3*
Leptin	BTA4	LEP	*LEP*
Osteopetrosis	BTA4	–	*SCL4A2*
Apoptosis peptide activating factor 1	BTA5	HH1	*APAF1*
Chondrodysplastic dwarfism	BTA6	BCD	*LBN*
Chondrodysplastic dwarfism	BTA6	–	*EVC2*
Caseins^a^	BTA6	CN	*CSN*
Calpastatin	BTA7	CAST	*CAST*
Umbilical hernia	BTA8	UH	–
Embryonic loss	BTA8	–	*SMC2*
Lactoglobulin	BTA11	LGB	*LGB*
Citrullinemia	BTA11	ASS	*ASS*
Cholesterol deficiency	BTA11	HCD	*APOB*
Prions	BTA13	PRNP	*PRNP*
Thyroglobulin	BTA14	–	*TG1*
Diacylglycerol acyltransferase	BTA14	–	*DGAT1*
Syndactyly	BTA15	Syndactyly	–
Maple syrup urine disease	BTA19	MSUD	*BCKDHA*
Slick hair	BTA20	–	*PRLR*
Slick hair	BTA23	–	*PRL*
Factor XI deficiency	BTA27	–	*FXI*
μ-Calpain	BTA29	–	*CAPN1*

*^a^Include CSN1S1, CSN2, CSN1S2, and CSN3*.

## Bovine Leukocyte Adhesion Deficiency (BLAD)

Although this autosomal recessive, eventually lethal condition was recognized in Holstein cattle, it is reported to be segregating in other cattle breeds where Holstein genetics were introduced (20). It is characterized by reduced expression of functional β_2_-integrins on all leukocytes (21). β_2_-integrins are adhesion proteins that are the primary effectors of neutrophil adhesion to receptors on endothelial cells of postcapillary venules and the subsequent egress of neutrophils through intercellular cell junctions into extravascular tissues to defend the host against normal flora and pathogens. Animals affected with this condition have abnormally low levels of β_2_-integrins on all leukocytes; however, the reduced expression on neutrophils produces inadequate innate immunity against microbes in all tissues. Animals affected with this condition show severe pneumonia, ulcerative gingivitis, periodontitis, papillomatosis, dermatophytosis, tooth loss, poor wound healing, and slow growth (1, 21–28). A single point mutation at position 383 in the transcribed RNA in the *CD18* gene was identified as the causative mutation. The substitution results in the replacement of an aspartic acid with a glycine at position 128 of the protein (D128G). *CD18* resides on chromosome 1 (29, 30). This mutation has been identified worldwide as responsible for the condition (31). A genetic test is available to identify carriers of the condition (29) and has been used to virtually eradicate the clinical condition from the Holstein breed within a period of not more than 5 years (32) without negatively impacting genetic merit for performance traits (33).

## Deficiency of the Uridine Monophosphate Synthase

This is an autosomal recessive lethal condition resulting from a deficiency of an enzyme that catalyzes the conversion of orotic acid to uridine-5′-monophosphate, which is the precursor of cytosine and thymine (components of DNA). Animals with this condition lack growth during embryonic development. Embryo mortality occurs approximately at 40 days of gestation (34). Carriers of this condition have a high incidence of return to estrus and long open day periods. The uridine monophosphatase synthase gene resides on chromosome 1 (35, 36). The substitution of a cytosine to a thymine at codon 405 of the gene has been identified as responsible for the condition (37), so it is possible to identify carriers of the condition.

## Bovine Chronic Interstitial Nephritis (BCIN)

This is a kidney condition characterized by interstitial fibrosis with inflammatory cell infiltration. This condition has been identified in the Wagyu, or Japanese Black Cattle. Animals with this condition have delayed growth and high levels of ureic nitrogen in blood (38). The gene resides in chromosome 1, and the causal mutation has been identified (39). Producers interested in producing export beef genetics using this breed, especially to the Japanese market, will be required to establish that breeding animals are not carriers of the condition.

## Horn Development

Horns may be a problem when handling cattle. It has been estimated that de-horning cattle, as well as loss of meat due to bruising from horns, represents a cost of approximately $25 million annually to the United States beef industry (40). Losses could be eliminated if carriers of alleles responsible for horn development are identified.

The locus of the gene responsible for horn development was detected on chromosome 1 and was termed POLL (40, 41). A locus has been recently identified on cattle chromosome 1 and is known in cattle and buffalo only. Wunderlich et al. (42) narrowed the region to a 2.5-Mb region on chromosome 1. Cargill et al. (43) identified 13 SNPs associated with horn development in this region, proposing several genes as responsible for the condition by sequencing a 1.6-kb region. Seichter et al. (44) identified nine additional SNPs in this region for the horn/polled condition. The gene from this locus is highly expressed in fetal tissue of horned animals, as compared with tissue from polled animals (45). It may be possible to select toward polled animals by using genomics information of horn development.

## Myostatin (*MSTN*)

Double muscling or muscle hypertrophy was recognized and documented in the nineteenth century (46). The locus that causes double muscling (mh locus) in cattle was localized on the telomeric end of chromosome 2 (12). At that time, the mh locus was being studied to identify the gene responsible for double muscling in cattle, a deletion in the transforming growth factor β in mice showed similar effects in mice (47). This protein was later named myostatin, and the symbol of the gene in the double muscling locus on chromosome 2 is *MSTN*. Later, it was demonstrated that this was the same gene that caused double muscling in cattle (48). After the identification of the gene responsible for double muscling, several studies were directed to identify SNPs associated with double muscling in cattle (13, 49, 50).

Growth and carcass traits were evaluated in double-muscled cattle before myostatin was identified. True double-muscled animals, or animals with two copies of the allele that produces double muscling, are heavier at birth (46). Casas et al. (51) found that double-muscled animals have up to 20% more calving difficulty than non-double-muscled breeds, but animals with only one copy of the *MSTN* allele increase muscling without having calving difficulty. From this, it can be concluded that use of true double-muscled animals can be problematic under rangeland conditions due to the need to assist the cow during calving. However, if this gene was to be used under rangeland conditions, it is necessary to correctly manage the herd to produce animals with one copy of the gene, which would increase muscle mass without calving problems, avoiding the production of double-muscled calves. Regarding carcass traits, Arthur (46) indicates that double-muscled animals have up to 30% more muscle mass than non-double-muscled animals. However, further studies detected an increase of 17% in muscle yield and 66% less fat in double-muscled animals (52). Meat of animals with double muscling has also been associated with tender meat (46, 53). Myostatin, as the gene that produces double muscling, can be considered in animal production to increase muscle mass, without increasing calving difficulty, only if managed in terminal crosses.

## Complex Vertebral Malformation (CVM)

Complex vertebral malformation is an autosomal recessive lethal condition of Holsteins observed in premature and mature calves (54). Congenital growth retardation, malformed vertebrae, and symmetric arthrogryposis typically characterize the condition although some morphological variations including various cardiac abnormalities occur (55, 56). Moreover, significant effects on reproductive performance and herd life have been reported (57). The causal point mutation (a G to T transversion) has been found to reside in an allele of a Golgi-resident transporter of UDP-*N*-acetylglucosamine encoded by *SLC35A3* on BTA3 that results in a valine substitution at position 180 with a phenylalanine (V180F) (58). This allele was carried by many of the same animals that carried BLAD, and the allelic frequency of the mutant allele had reached as high as 20–30% in many countries before a rapid genetic diagnostic test was available to prevent further transmission of this mutant allele (58).

## Leptin (*LEP*)

Leptin is the hormone produced by the obesity gene (ob). It is secreted by adipocytes, and it has been associated with feed consumption and energy balance in mice and humans. The leptin gene is located on bovine chromosome 4. A genetic marker was identified in the sequence (59) and in the promoter region of the bovine gene (60). Buchanan et al. (59) proposed that the missense mutation in the gene sequence could be considered the causative mutation in differences of fat deposits in cattle. Barendse et al. (61) indicated that there is no association between genetic markers in the leptin gene with fat in cattle; however, additional studies have supported the theory that different alleles of the gene are associated with differences in fat in cattle (60, 62, 63). The use of genetic markers in the leptin gene could be of use in beef production.

## Osteopetrosis

The condition osteopetrosis was identified in cattle, which is also known as marble bone disease. Animals are characterized by forming exaggeratedly dense bones. This is the result of a deficiency in the number, or lack of function, of osteoclasts (64). Animals with this condition are usually stillborn, slightly premature with small body size. They often display distinctive features in the skull like flat skull, impacted molars, shortened mandibles, and protruding tongue (65, 66). A deletion in the *SCL4A2* gene has been associated with the condition in Red Angus. The gene resides on chromosome 4 (64).

## Apoptosis Peptide Activating Factor 1 (*APAF1*) Truncation

A haplotype named HH1 was initially identified on BTA5 using high-density SNP genotyping and associated with a decrease in conception rates and an increase in stillbirths in Holstein cattle (67). The causal mutation has now been identified to be a result of a truncation of the APAF1 protein and has been traced to the bull Pawnee Farm Arlinda Chief (Chief), a bull born in 1962 that sired several other prominent bulls used for artificial insemination, some of which have since had their genomes sequenced (68, 69). Through the use of artificial insemination, Chief also produced over 16,000 daughters, 500,000 granddaughters, and 2 million great-granddaughters (69). Owing to the widespread use of his genetics, the estimated cumulative number of spontaneous abortions caused by APAF1 truncation over the three decades that Chief’s alleles became highly frequent to be more than 100,000 in the United States and nearly 500,000 worldwide.

## Chondrodysplastic Dwarfism

Dwarfism has been studied in cattle and other species. The principal characteristic of this condition is the abnormal bone ossification of extremities. It has also been associated with other conditions in the animal. Different genes produce dwarfism in different species. Chondrodysplastic dwarfism has been identified in the Japanese Brown Cattle. Economic losses can be attributed to this condition if the breed is used in breeding schemes. The locus responsible for this condition was identified on chromosome 6 (70). Two different DNA changes were identified in the LIMBIN gene associated with dwarfism. The first was a mutation that produces an alternative splicing site in the gene, and the other was a deletion in the gene (71). Takeda et al. (71) proposed these changes in the gene as responsible for chondrodysplastic dwarfism in Japanese Brown cattle.

Genetic variants in the Ellis van Creveld Syndrome 2 gene have also been proposed as responsible for chondrodysplastic dwarfism in Tyrolean Grey cattle (72). This gene also resides on chromosome 6. A 2-bp deletion was identified as responsible for the condition. The deletion produces a premature stop codon and thus a loss of function in the protein (72). Identification of mutations for the chondrodysplastic dwarfism allows genetic testing with the objective to eliminate this condition in cattle.

## Caseins (*CSN*)

Milk proteins in ruminants have been comprehensively studied due to their importance in milk composition and cheese-making properties (73, 74). Dalgleish and Corredig (74) have described in detail the structure of the casein micelles and its changes during the processing of milk. Farrell et al. (73) reported the current nomenclature of the proteins of the cow in milk, with emphasis on caseins. Polymorphisms identified in the genes of caseins in bovine have also been summarized (75). Caseins comprise 80% of total proteins in milk.

There are four casein molecules produced (Alpha_S1_, Alpha_S2_, Beta, and Kappa). These proteins are coded by four genes on chromosome 6. The order of these genes on the chromosome is *CSN1S1, CSN2, CSN1S2*, and *CSN3*. The genomic region is referred to as the CN locus because these four genes are tightly linked (74, 75). Of particular interest in cheese production has been the kappa-casein genetic variants. Farrell et al. (73) have described in detail the genomic differences among variants in each casein gene. The B variant or allele is of particular interest because it has been associated with increased cheese production (76). Differences between the A and the B alleles from the kappa-casein protein is the substitution of an isoleucine for a threonine at position 136 and the substitution of an alanine for an aspartic acid in position 148 of the protein (73). SNPs associated with variants in the casein genes have been developed (77).

Using high-throughput genotyping technology, additional associations for casein production have been identified. A GWAS for bovine milk caseins and lactalbumin was done in Dutch Holstein Friesian cows (78). Several genomic regions were associated with the proportion of different caseins in milk. Schopen et al. (78) concluded that the proportion of genetic variance explained by the SNP on chromosomes 6, 11, and 14 could be explained by the casein locus on chromosome 6, beta-lactoglobulin on chromosome 11, and diglyceride acyltransferase-1 (DGAT1) on chromosome 14. Gambra et al. (79), performing a GWAS in a Holstein X Jersey population, identified six additional SNP, on chromosome 6, associated with caseins in milk. Given the proximity of the casein genes in a single locus, it has been suggested that SNP in the casein genes can be used in haplotypes instead of selecting each variant independently (75, 77). Different alleles may be suitable for management to increase milk production or to increase cheese production in dairy production systems.

## Calpastatin (*CAST*)

Meat tenderness is one of the most important factors for consumer satisfaction. The calpastatin proteolytic axis has been identified as an important process to established meat tenderness. Calpastatin is the regulator of m-Calpain and μ-Calpain. m-Calpain and μ-Calpain are proteolytic enzymes responsible for the breakage of muscle fibers, producing postmortem tenderization of meat (80). Calpastatin is the natural inhibitor of calpains in this proteolytic system.

The gene that produces calpastatin (*CAST*) is located in bovine chromosome 7, and a genetic marker was identified within this gene. An association between meat tenderness and this genetic marker in the calpastatin gene has been observed in several studies (81, 82). This makes calpastatin a suitable gene to develop genetic markers associated with meat tenderness in other breeds, especially for breeds with known tough meat.

## Umbilical Hernia

Umbilical hernia is a bovine defect observed after birth. It consists of the protrusion of the intestine or other organs through the abdominal wall at the umbilicus. In Israeli Holstein populations, frequency of retained placenta increased 7% in first-parity cows without umbilical hernia, compared with 18% in first-parity cows with umbilical hernia (83).

The locus responsible for umbilical hernia resides on chromosome 8. The gene is still unidentified, but the gene resides within an 8-Mb region (83). Genomics can be of assistance in identifying the gene and the causative mutation for the condition. Marker-assisted selection could be used to select against umbilical hernia to reduce costs associated with placenta retention.

## Embryonic Loss

Fertility is an important factor that affects the dairy industry. The ability to produce offspring is a major component of milk production. Embryo loss hampers milk production for the cow, and it is an important economic factor in the dairy industry. A locus on chromosome 8 has been identified as responsible for death loss in Holsteins. Fritz et al. (84) identified that the Holstein Haplotype 3 (HH3) was responsible for embryo losses in French Holstein. Daetwyler et al. (85) had the same conclusion when evaluated HH3 using information from the 1,000 Bull Genomes Project.^1^ The condition was identified by the lack of homozygous individuals for a particular haplotype in the United States Holstein population. The mutation causing embryonic loss was estimated to reside in the Structural Maintenance of Chromosomes Protein-2 (SMC2) gene on chromosome 8. McClure et al. (17) confirmed the effect of the haplotype, within the SMC2 gene, as responsible for embryonic loss in Holstein.

## Lactoglobulin (*LGB*)

The beta-lactoglobulin is the major protein in whey. Although LGB is not implicated in the coagulation process of milk, different variants of this gene affect renneting properties of raw milk. The *LGB* gene resides on chromosome 11, and 11 different variants have been described. The most common variants are alleles A and B in most dairy breeds; however, allele C is common in Jersey, and alleles D and E are common in other breeds (73, 75). The sequence of the variant B for this protein is considered the standard. Differences between variants B and A are the change of a glycine for an aspartic acid in position 64 and the substitution of an alanine for a valine in position 118 of the protein (73). Variants A and B have different properties affecting milk. The B variant of this protein denatures faster than variant A; therefore, heat stability is higher for the latter variant (86). Jakob and Puhan (86) indicate that kappa-casein reacts faster with the B variant, when compared to the A variant of LGB. SNP in the gene that produces the protein can be used to improve cheese production.

## Citrullinemia

This autosomal recessive lethal genetic condition is characterized by high levels of ammonia in blood (87). This is due to a deficiency in activity of the enzyme argininosuccinate synthase that is produced by the gene *ASS1*. The enzyme is a key component of the urea cycle (1, 34). Animals with this condition are unable to excrete ammonia and have neurologic symptoms, producing perinatal mortality. The gene resides on chromosome 11, and the gene has been identified. The initial report demonstrated the first use in animal production of the polymerase chain reaction (PCR) combined with an endonuclease enzyme (*Ava*II) digestion to identify a restriction fragment length polymorphism of a fragment of the gene to identify carriers of the condition (88). Current technology would be capable to identify the SNP responsible for the condition.

## Cholesterol Deficiency in Cattle

A recessive condition has been recently identified in Holstein cattle. Calves show severe hypocholesterolemia and die soon after birth due to diarrhea (89). Kipp et al. (89) identified a region of chromosome 11 associated with this condition on chromosome 11. The analysis of the pedigree of calves, known to have died from this condition, where traced to a predominant Canadian Holstein bull (Maughlin Storm). It was concluded that he was a carrier of the condition. Further analysis of this condition was pursued by Menzi et al. (90). An insertion of 1.3 kb of a transposable long terminal repeat (LTR) element within exon 5 of the *APOB* gene was identified (90). The LTR element identified is a vestige of viral DNA inserted in the host. These elements have also been identified in human (91). The insertion results in a frameshift that starts at amino acid 135 and produces a 97% truncation of the 4,567 amino acid long apolipoprotein B (90). Results from Menzi et al. (90) can be used as a genetic test to identify carriers of the condition in Holstein.

## Prion (*PRNP*) Diseases

Bovine spongiform encephalopathy (BSE) is a neurodegenerative fatal condition affecting cattle. This condition is a transmissible spongiform encephalopathy that is similar to scrapie in sheep, chronic wasting disease in deer, and Creutzfeld–Jacob disease (CJD) in humans. BSE is caused by an accumulation of an abnormally folded isoform of the prion protein in central nervous system tissues. While the vast majority of cases of BSE were the result of ingesting feed derived from animal origin protein products contaminated with infected central nervous system tissues, today there are recognized atypical BSE cases that are thought to arise spontaneously and are not attributed to a transmissible origin in feedstuffs (92). Defined by their atypical molecular profiles on western blots when compared with classical BSE, atypical BSE cases tend to occur in older cattle than what is seen with classical BSE. One study of *PRNP* haplotypes from six atypical BSE cases has suggested a genetic determinant in or near *PRNP* influencing susceptibility of cattle to atypical BSE (93). Although believed to be extremely rare (94), one H-type BSE case (95) was associated with a heritable E211K mutation in the prion protein gene (*PRNP*) and is now thought to represent the bovine ortholog of the most common form of genetic CJD (E200K) in humans (96). Intracranial inoculation of infected brain material from the E211K BSE case into cattle possessing the 211K allele has demonstrated a very rapid onset of clinical BSE, consistent with a genetic form of BSE (97). A comprehensive review is available (98). The locus of the prion protein resides on chromosome 13 and consists of three exons. Initial studies identified three gene variants, differing in the number of repeats for eight peptides. Five, six, and seven repeats of the peptides were identified (99, 100). Seabury et al. (101), besides evaluating the repeats, studied the promoter region without identifying an association. However, there were differences in allelic frequencies in the intronic region of the gene. Additional studies have identified two indels in the bovine *PRNP* promoter region that have been studied for their association with BSE (102–104). Others examined the prevalence of the indel polymorphisms among selected cattle populations and found no association with the development of experimentally transmitted TSEs in cattle (105–107); this provided evidence that genetic factors associated with resistance to classical BSE in cattle do not provide resistance to cattle naturally infected with atypical BSE, thus suggesting that atypical BSE progresses *via* an alternative pathogenesis route compared to classical BSE and therefore is most likely a spontaneous prion disease in cattle.

## Thyroglobulin (*TG1*)

The thyroglobulin gene (*TG1*) resides on chromosome 14. The first association of genetic markers in this gene was reported by Thaller et al. (108). Since then, several studies have established the association of this gene with intra- and extra-muscular fat in *Bos taurus* (109, 110). Although fat could be considered a secondary trait in beef production, the use of genetic markers for this trait could be of value in evaluating marbling in carcasses of *B. taurus* or *Bos indicus* origin.

## Diacylglycerol Acyltransferase (*DGAT1*)

The gene that produces this protein is known as *DGAT1*, which is localized on chromosome 14, neighboring the *TG1* gene (108, 111). This marker was originally associated with fat yield in milk (111), and additional studies have validated this association (112). This genetic marker has also been associated with marbling and fat thickness in beef cattle (108, 113, 114). This genetic marker residing in this gene is associated with fat production in dairy and beef cattle.

## Syndactyly

This genetic condition is also known as “Mule Foot.” It is characterized by the fusion or stenosis of the functional phalanges in the bovine. The condition precludes natural mount in sires. This genetic condition presents susceptibility to hyperthermia due to high environmental temperatures. The locus resides on chromosome 15; however, the causative gene or change in the DNA has not been identified (115). Microsatellite markers in the neighboring region have been used to identify carriers of the condition (116). The *LRP4* gene has been proposed as the candidate for the condition in this chromosomal region, and SNPs in the gene have been associated with the condition (117).

## Maple Syrup Urine Disease

Animals with this condition have sweet-smelling urine. It is a progressive neurological condition, resulting in the inability of the animal to walk and ultimately results in death. The condition is the result of a mutation in the *BCKADH* gene. The enzyme has four subunits (Ea-alpha, E1-beta, E2, and E3). A mutation in the E1-alpha subunit, from a cytosine to a thymine, produces an incomplete enzyme (changes from a glutamine to a stop codon) in Hereford cattle. Animals with this condition have elevated levels of isocaproic acid which is the substrate of the enzyme (118, 119).

The Pre-E1-alpha subunit of the branched chain alpha-ketoacid dehydrogenase gene resides in chromosome 19. The mutation in Hereford was first identified, and further studies established that a different mutation was responsible for the condition in Shorthorn (118–120). Additional studies are needed to determine the causative mutation in Shorthorn.

## Slick Hair

This condition should be of utmost importance for milk production under tropical and subtropical conditions. Slick hair coat has been observed in tropical breeds of *B. taurus* and has been studied in the Senepol and Carora breeds. These animals have very short, slick hair coats (121). Although it is unknown where the condition originated, it is known that Criollo cattle in the Americas possess this condition. The capability to preserve normal body temperature during heat stress conditions is an important trait in tropical and subtropical cattle. Heat stress is a problem for animals not adapted to tropical conditions, negatively impacting milk production. Senepol cattle with slick hair are capable of maintaining a lower body temperature when compared with Senepol without this condition. Senepol cattle are known to be as heat-tolerant as Brahman cattle (121, 122). Mariasegaram et al. (123) reported that the locus responsible for slick hair resided on chromosome 20. Further studies confirmed that the slick hair locus resides on chromosome 20 (124). Dikmen et al. (124) indicate that three potential candidate genes are in the region where the slick hair locus resides (*SKP2, SPEF2*, and *PRLR*). Littlejohn et al. (125) propose mutations in the prolactin receptor (*PRLR*) on chromosome 20 and the prolactin gene (*PRL*) on chromosome 23, as responsible for the slick hair condition. Littlejohn et al. (125) indicate that animals with the slick hair condition produced more milk than animals without the condition. Furthermore, Dikmen et al. (124) show that Holstein cattle with the slick hair condition produce more milk than Holstein cattle without the condition. This condition in dairy cattle under tropical conditions should be of economic importance if introduced.

## Factor XI Deficiency

First reported in 1975 (126), Factor XI deficiency in Holstein cattle was later shown by planned matings to be inherited as an autosomal recessive trait (127). Factor XI is a plasma serine protease critical for activation of the intrinsic blood coagulation cascade. Factor XI-deficient cattle can be asymptomatic or exhibit symptoms including prolonged bleeding times following injections or insect bites, production of bloody milk and anemia. Although first reported in an 8-year-old steer, there are reports of lower calving and survival rates, and increased susceptibility to infectious diseases (128). The initial estimate of heterozygote frequency based upon activated partial thromboplastin times ranged between 8 and 17% (127). The mutation causing Factor XI deficiency is a 76-bp insertion within exon 12 that introduces a premature stop codon, thus resulting in a truncated Factor XI protein that is missing the functional serine protease domain responsible for proteolytic activation of Factor XI (129). Limited conscientious testing of bulls entering artificial insemination programs through the use of the activated partial thromboplastin time test reduced the allelic frequency of the mutant allele to 1.2% among 419 animals genotyped from the Dairy Bull DNA Repository at the time the DNA mutation was identified and a DNA PCR diagnostic test was available (129).

## μ-Calpain (*CAPN1*)

This protein is a component of the calpastatin proteolytic axis. Its gene is located on chromosome 29, and the gene symbol is *CAPN1*. The first SNP identified in this gene was associated with meat tenderness in *B. taurus* breeds (130). Additional SNP were developed and found to be of better use than the original in *B. indicus* cattle (81, 131–133). Currently, there are several efforts to identify SNP associated with meat tenderness in beef cattle from *B. indicus* origin (131, 134, 135) and other native breeds (136). There are also attempts to associate SNP of *CAPN1* with meat quality traits (137). Genetic markers in CAPN1 are suitable to be used by producers in Latin America to increase meat quality.

## Final Remarks

Relevant information regarding genetic conditions affecting productivity and health of cattle is available. Such is the case of the Quantitative Trait Loci Data Base, or QTLdb,^2^ which maintains current information of genomic regions that have been associated with traits of economic importance in cattle. The database contains information for 81,652 quantitative trait loci detected in the cattle genome. Information includes information for health, meat and carcass, milk production, growth, reproduction, and exterior traits of cattle (138). Similarly, the Online Mendelian Inheritance in Animals^3^ currently contains a list of 494 cattle genetic traits or disorders, of which 229 are known to be inherited in a Mendelian fashion. There are 130 traits or disorders of which the point mutation, or quantitative trait nucleotide, is known. In the current database, there is information for 181 traits in which cattle can be used as a model for humans (139). These sources are readily available to cattle producers, students, and researchers in the field of cattle genomics.

Recognition of genetic conditions is an important component in animal production. Screening for genetic conditions needs to be assessed by the producer, according to the production system and breeds used. Several genetic conditions would improve productivity, while others would be deleterious. For each production system, it is necessary to screen the herd for known genetic conditions.

There are genetic conditions that would improve productivity (i.e., slick hair). For these genetic conditions, it would be possible to select for favorable alleles within the herd. This would increase productivity and make the production system more efficient. This is important where productivity is limited by hazardous environmental conditions.

The producer may want to introduce genetic conditions in the herd, or completely avoid them (i.e., myostatin). These genetic conditions need to be carefully assessed if they were to be introduced in the herd. It would increase productivity by increasing amount of muscle mass and salable meat. However, adequate management of the herd needs to take place to avoid detrimental effects that would result in economic losses to the producer. These losses would be in the form of expenses due to calving difficulty, or losses of the calf due to poor management.

If the goal is to sell to international markets, there are genetic conditions that producers need to be aware (i.e., BCIN). These conditions have only been identified in specific breeds, and they would not represent a problem in other breeds. However, if the producer decides to introduce these breeds for international trade, screening for genetic conditions needs to be undertaken before productivity is hindered.

Deleterious genetic conditions need to be recognized (i.e., syndactyly). These genetic conditions will limit productivity of animals expressing the trait. Animals should be eliminated from the herd to benefit the production system. A point of caution is that in spite of current genetic testing being highly effective at accurately identifying carrier animals of particular traits, leukochimerism can result in erroneous test results if blood is used as a convenient DNA source; test results must always be confirmed with an independent tissue source not subject to mixed genotypes (140).

As advances in molecular biology continue, the impact on our ability to more rapidly detect traits of economic importance will only improve. Use of haplotype reconstruction and haplotracking with a database of >1 million cattle^4^ will enable discovery of various traits (many of which may be unrecognized) that impact livestock performance and production.

## Author Note

Mention of trade name, proprietary product, or specified equipment does not constitute a guarantee or warranty by the USDA and does not imply approval to the exclusion of other products that may be suitable.

## Author Contributions

EC and MK wrote and reviewed the manuscript.

## Conflict of Interest Statement

The authors declare that the research was conducted in the absence of any commercial or financial relationships that could be construed as a potential conflict of interest.

## Footnotes

^1^www.1000bullgenomes.com.

^2^http://www.animalgenome.org/cattle/maps/db.html.

^3^http://omia.angis.org.au/.

^4^http://aipl.arsusda.gov/reference/recessive_haplotypes_ARR-G3.html.
